# Protection from septic peritonitis by rapid neutrophil recruitment through omental high endothelial venules

**DOI:** 10.1038/ncomms10828

**Published:** 2016-03-04

**Authors:** Konrad Buscher, Huiyu Wang, Xueli Zhang, Paul Striewski, Benedikt Wirth, Gurpanna Saggu, Stefan Lütke-Enking, Tanya N. Mayadas, Klaus Ley, Lydia Sorokin, Jian Song

**Affiliations:** 1 Institute of Physiological Chemistry and Pathobiochemistry, University of Muenster, Muenster 48149, Germany; 2 Cells-in-Motion Cluster of Excellence, University of Muenster, Muenster 48149, Germany; 3 Department of Nephrology and Rheumatology, University of Muenster, Muenster 48149, Germany; 4 Institute for Computational and Applied Mathematics, University of Muenster, Muenster 48149, Germany; 5 Center for Excellence in Vascular Biology, Department of Pathology, Brigham and Women's Hospital and Harvard Medical School, Boston, Massachusetts 02115, USA; 6 Division of Inflammation Biology, La Jolla Institute for Allergy and Immunology, La Jolla, California 92037, USA

## Abstract

Acute peritonitis is a frequent medical condition that can trigger severe sepsis as a life-threatening complication. Neutrophils are first-responders in infection but recruitment mechanisms to the abdominal cavity remain poorly defined. Here, we demonstrate that high endothelial venules (HEVs) of the greater omentum constitute a main entry pathway in TNFα-, Escherichia coli (E. coli)- and caecal ligation and puncture-induced models of inflammation. Neutrophil transmigration across HEVs is faster than across conventional postcapillary venules and requires a unique set of adhesion receptors including peripheral node addressin, E-, L-selectin and Mac-1 but not P-selectin or LFA-1. Omental milky spots readily concentrate intra-abdominal E. coli where macrophages and recruited neutrophils collaborate in phagocytosis and killing. Inhibition of the omental neutrophil response exacerbates septic progression of peritonitis. This data identifies HEVs as a clinically relevant vascular recruitment site for neutrophils in acute peritonitis that is indispensable for host defence against early systemic bacterial spread and sepsis.

## Abstract (web summary)

Neutrophils are critical in preventing the transition of acute peritoneal infection to sepsis. Here the authors show in three mouse models of peritonitis that neutrophils enter the abdominal cavity via high endothelial venules of the greater omentum, and characterize adhesion molecules involved.

## Graphical abstract

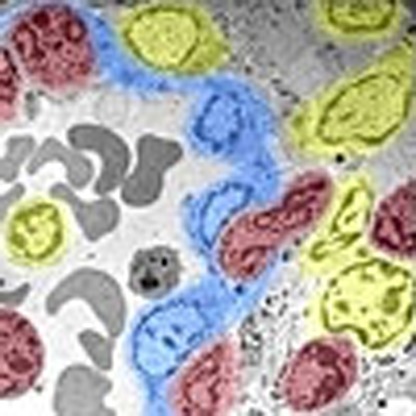

Multiple clinical conditions such as trauma, surgery, liver cirrhosis, peritoneal dialysis catheters or disruption of the gastrointestinal tract can cause peritonitis. Subsequent systemic spread of intra-abdominal pathogens results in sepsis with high mortality and medical treatment is challenging^1^.

Leucocyte influx into the peritoneal cavity is a hallmark of abdominal inflammation but the routes of neutrophil emigration remain enigmatic. Here we studied the greater omentum, an intraperitoneal organ deriving from the greater curvature of the stomach and the spleen. It consists of a double layer of mesothelial cells that encapsulates a highly vascularized parenchyma of fatty tissue and multiple immune cell aggregates called milky spots. These are considered unconventional secondary lymphoid organs^2^ and consist mostly of B1-cells, macrophages, T cells and mast cells, and lack follicular dendritic cells and germinal centres^3^,^4^. Milky spots dynamically increase in number and size in chronic inflammation or irritation^5^,^6^ and a role in natural antibody production and adaptive peritoneal immunity is well established^2^,^7^,^8^. A key feature is the presence of a specialized type of postcapillary venule called high endothelial venule (HEV) that is essential for lymphocyte trafficking^9^. Similar innate B1-cell-rich clusters in adipose tissues have been observed in the mouse mesentery, the pleural cavity and the pericardium, and are termed fat-associated lymphoid clusters^10^,^11^.

To date, little is known about the regulatory functions of the greater omentum in innate immunity. Therefore, we studied its impact on leucocyte recruitment to and bacterial spread from the abdominal cavity in murine models of acute peritonitis. We can show that the first wave of neutrophils recruited to the inflamed peritoneum uses milky spot HEVs as exit site with unique molecular properties and that specific inhibition of this pathway leads to systemic bacterial spread and clinical exacerbation of septic peritonitis.

## Results

### Omental HEVs as neutrophil exit route in peritonitis

**Figure 1:**
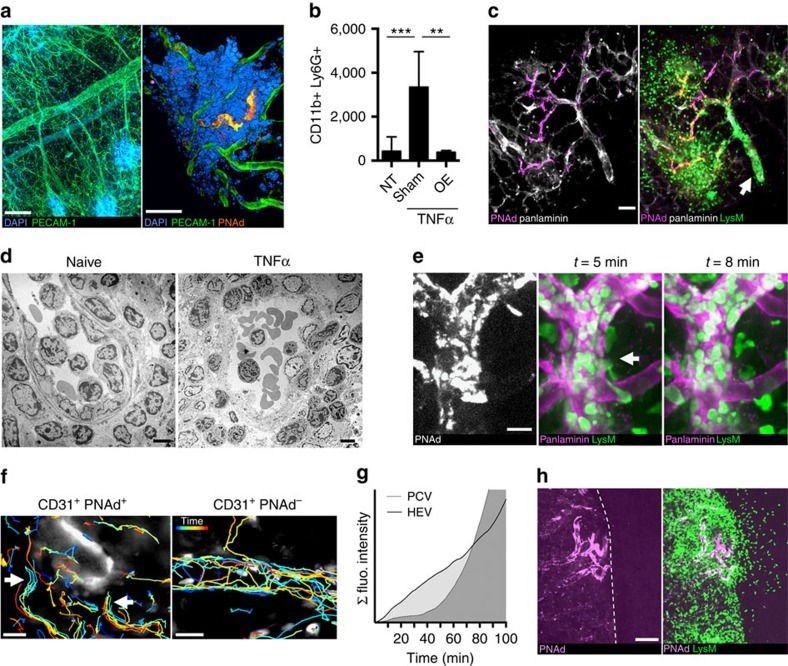
HEVs of the greater omentum enable rapid neutrophil exit in TNFα-peritonitis. (**a**) Confocal micrographs of the naive omentum show vascularization and milky spots (left panel). PNAd^+^PECAM-1^+^-HEVs are distinct vascular compartments within the milky spot (right panel). Scale bars, 120 μm (left) and 40 μm (right). (**b**) Non-treated (NT), omentectomized (OE) or sham-operated mice were compared by analysing the peritoneal lavage after 60 min i.p. treatment with TNFα. Cell number as mean±s.d. *n*=5 per condition of three independent experiments. Student's *t*-test **P<0.01, ***P<0.001. (**c**) Confocal micrograph of an inflamed omentum of a *LysM-eGFP* reporter mouse after 60 min i.p. TNFα treatment. Panlaminin is used as a vessel marker. Note the luminal accumulation of GFP^bright^ neutrophils in the PNAd^−^-postcapillary venule (arrow). Scale bar, 40 μm. Representative of five independent experiments. (**d**) Transmission electron micrographs of naive and TNFα-inflamed milky spot HEVs. Scale bar, 5 μm. Representative of >10 milky spots in two independent experiments. (**e**) Representative snapshots from a 4D intravital spinning disc microscopy video with PNAd- and panlaminin staining using a *LysM-eGFP* mouse (video 3). The arrow indicates extravasating GFP^bright^ neutrophils. Scale bar, 20 μm. (**f**) Video recordings of PNAd^+^ HEVs and PNAd^−^ PCV were used for *LysM-eGFP* neutrophil tracking 60 min after TNFα application. Colour-coding of tracks indicates time (20 min). HEV stained by MECA-79 in white. Arrows indicate active neutrophil transmigration sites. Representative of three independent experiments. Scale bar, 20 μm. (**g**) Analysis of an intravital recording of a milky spot HEV and a conventional PCV in the same field of view over a time course of 100 min in a *LysM-eGFP* mouse. TNFα was topically applied at 0 min and the integral of the pixel intensity as a measure of leucocyte accumulation in the region of interest was plotted over time. Data representative of four milky spots in two independent experiments. (**h**) Intravital microscopy of the inflamed exteriorized omentum in *LysM-eGFP* mice with PNAd staining. The dashed line marks the omental border. Scale bar, 100 μm. Representative of two independent experiments.

Milky spots are located predominantly at the lateral border of the greater omentum and peripheral node addressin (PNAd) as key marker for HEVs can be detected using the MECA-79 monoclonal antibody (Fig. 1a; Supplementary Fig. 1a)^9^. At steady state, there are few neutrophils present in the omentum or the abdominal cavity, but neutrophil influx can be provoked by intraperitoneal application of TNFα (Fig. 1b)^12^. Omentectomized mice showed a strong reduction in peritoneal neutrophil influx at 1 h after TNFα application indicating a functional role of the greater omentum in acute peritonitis (Fig. 1b). Using *LysM-eGFP* reporter mice in which neutrophils are brightly fluorescent^13^ with 1 h intraperitoneal (i.p.) TNFα treatment, we observe that neutrophil invasion is first triggered in milky spots in close vicinity to HEVs (Fig. 1c). Electron microscopy revealed polymorphonuclear cells in close contact to lymphocytes at luminal, subendothelial and perivascular sites in inflamed milky spots (Fig. 1d) suggesting a similar exit site for both leucocyte subsets.

Next, we developed a surgical technique to perform intravital microscopy on the omental microcirculation (Supplementary Fig. 1b) showing early neutrophil–endothelium interactions in milky spots and parenchymal neutrophil migration adjacent to HEVs in *LysM-eGFP* mice (Supplementary Movies 1 and 2; Supplementary Fig. 1c). Intravital 4D spinning disc confocal microcopy confirmed HEVs as predilection site for active neutrophil transmigration in acute TNFα-induced inflammation (Fig. 1e, Supplementary Movie 3). A similar phenotype was observed in a neutrophil-specific *Ly6G-tdTomato* reporter mouse^14^ (Supplementary Movie 4).

We noticed that at early (1 h) but not later stages (>3 h) of TNFα-inflammation neutrophils accumulated intraluminally in PNAd^−^/PECAM-1^+^ postcapillary venules (PCV; Fig. 1c; Supplementary Fig. 2a). Tracking of neutrophil motion 60 min after TNFα revealed distinct HEV exit ramps as described for lymphocytes^15^, whereas PCVs predominantly showed rolling neutrophils (Fig. 1f). Time-lapse video recordings indicate a different temporospatial response of neutrophil recruitment in HEVs compared with PCVs with the former being dominant in the first 60–80 min of TNFα-inflammation (Fig. 1g; Supplementary Fig. 2b; Supplementary Movie 5). The first wave of neutrophil egress out of the omental parenchyma into the surrounding area is initiated in close vicinity to milky spot HEVs as shown by intravital microscopy (Fig. 1h). Together, this data indicates that HEVs constitute a neutrophil exit platform in the early phase of peritoneal inflammation allowing rapid neutrophil entry into omental milky spots and subsequent release into the abdominal cavity.

### Unique requirement of adhesion molecules

**Figure 2:**
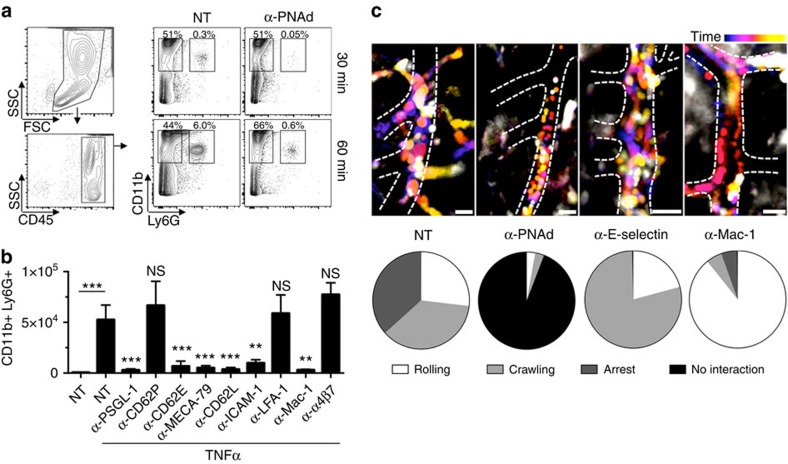
Unique adhesion receptor requirements of HEV-mediated neutrophil recruitment. (**a**) Flow cytometry gating strategy and analysis of the peritoneal lavage after 30 or 60 min of TNFα-induced peritonitis with and without (NT, non-treated) PNAd inhibition by high-dose i.v. MECA-79 mAb injection. Data representative of two independent experiments. (**b**) The effect of different blocking antibodies on neutrophil accumulation in the abdominal lavage was evaluated after 1 h TNFα treatmensing flow cytometry as in (a). *n*=6 of least three independent experiments. Data as mean total cell number±s.d. Statistical significance is indicated relative to NT with TNFα. Student's *t*-test **P<0.01, ***P<0.001. (**c**) To investigate the luminal neutrophil behaviour, E-selectin, L-selectin or Mac-1 was blocked by i.v. injection of monoclonal antibodies and intravital microscopy of inflamed omental milky spots was performed in *LysM-eGFP* mice at high magnification. Time is colour-coded and visualizes cell motion within 3–5 min duration. Dashed lines indicate vessel borders. Scale bar, 20 μm. Quantification of intraluminal neutrophil behaviour in HEVs shown as pie charts for each condition. Cell numbers for rolling/crawling/arrest/time without any neutrophil–endothelium interaction in seconds: NT *n*=25/33/34/0, α-PNAd=1/1/0/210, α-E-selectin *n*=9/34/0/0 and α-Mac-1 *n*=34/2/2/0.

To delineate the molecular pathway of HEV-mediated neutrophil recruitment, we blocked adhesion molecules by monoclonal antibodies. Flow cytometry showed a population of CD11b^+^Ly6G^+^ neutrophils in the abdominal cavity already within 30 min and a further increase at 60 min after TNFα (Fig. 2a). PNAd blockade by 50 μg MECA-79 mAb abolished most neutrophil influx, but did not affect CD11b^+^Ly6G^−^ macrophages (Fig. 2a). Likewise, we found an absolute requirement for L-selectin (CD62L), E-selectin (CD62E), P-selectin glycoprotein ligand-1 (PSGL-1), CD11b/CD18 (Mac-1) and intercellular adhesion molecule 1 (ICAM-1), whereas P-selectin (CD62P), α_4_β_7_-integrin and CD11a/CD18 (LFA-1) did not play a significant role as assessed at the 60 min time point (Fig. 2b). A similar effect of blocking antibodies on neutrophil recruitment was determined in omental extracts (Supplementary Fig. 3a) and in response to intraperitoneal bacterial lipopolysaccharide (Supplementary Fig. 3b). We confirmed the unexpected role of Mac-1 using knockout mice^16^ showing reduced neutrophil numbers in TNFα-inflamed omenta compared with wild-type controls (Supplementary Fig. 3c).

Intravital imaging indicated that intraluminal neutrophils exhibited rolling, crawling and arrest in inflamed HEVs (Fig. 2c). Blocking concentrations of MECA-79 mAb abolished most neutrophil-HEV interactions (Fig. 2c; Supplementary Movie 6). E-selectin can be detected in TNFα-exposed HEVs using immunofluorescence microscopy (Supplementary Fig. 3d) and its inhibition induced a prominent crawling phenotype (Fig. 2c; Supplementary Movie 7). Mac-1 blockade prevented arrest and crawling, leaving only rolling neutrophils (Fig. 2c; Supplementary Movie 8).

### Inhibition of HEV-mediated neutrophil exit aggravates sepsis

**Figure 3:**
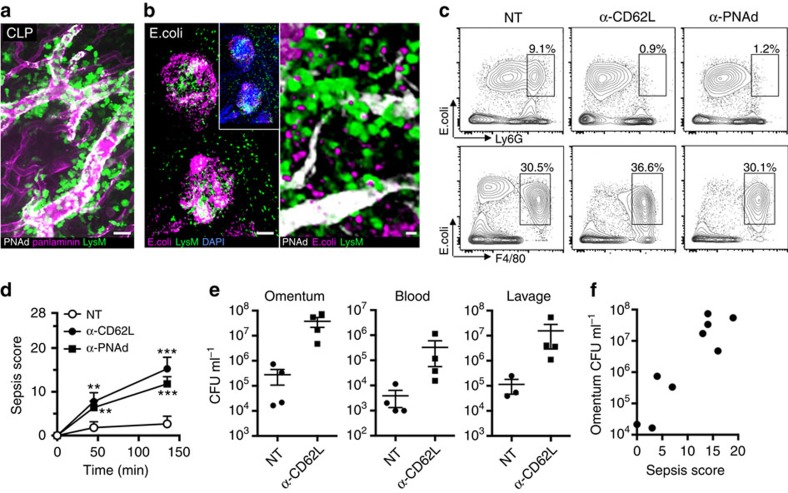
Bacterial peritonitis triggers HEV-mediated neutrophil recruitment required to prevent septic peritonitis. (**a**) Polymicrobial sepsis was induced by caecal ligation and puncture and the greater omentum was imaged by confocal microscopy 4 h after surgery. Sham control did not show major signs of inflammation (Supplementary Fig. 4a). Representative of three independent experiments. Scale bar, 30 μm. (**b**) Fluorescently labelled E. coli were injected i.p. and confocal micrographs of the omentum were taken after 2 h. The left panel shows a low magnification image of two milky spots. The right panel shows *LysM-eGFP* neutrophils adjacent to HEVs and labelled E. coli. Scale bars, 100 μm (left) and 10 μm (right). (**c**) Flow cytometry data of the peritoneal lavage showing colocalization of fluorescently labelled E. coli and CD11b^+^F4/80^+^ macrophages or CD11b^+^Ly6G^+^ neutrophils 2 h after infection in untreated (NT, non-treated), L-selectin- or PNAd-blocked mice. Similar data has been obtained for omental extracts (Supplementary Fig. 4c). Data is representative of two independent experiments. Gating strategy on CD45^+^CD11b^+^ cells in Supplementary Fig. 4b. (**d**) Clinical evaluation of mice i.p. injected with live E. coli using a murine sepsis score with a maximum of 28 points^17^. PBS-treated (NT, *n*=6), blocking anti-L-selectin antibody (*n*=4) or blocking anti-PNAd antibody treated (*n*=5) mice were compared over a 2-h time course in 2–3 independent experiments. Data shows mean±s.d. Significance shown compared with untreated condition. Two-way analysis of variance with Bonferroni posthoc correction. **P<0.01, ***P<0.001. (**e**) The abdominal lavage, the greater omentum and blood was collected after 2 h E. coli peritonitis for the determination of colony forming units (CFUs). Data shows mean±s.d. (**f**) Correlation of omental E. coli bacterial load and the maximal clinical sepsis score obtained during 2 h after infection. Each data point represents one mouse of the untreated or anti-L-selectin treated groups at the 120 min time point.

Our data suggested that the greater omentum may play a role in the early innate immune response of bacterial infections. We used a sub-lethal caecal ligation and puncture (CLP) model to induce polymicrobial peritonitis and found that substantial neutrophil recruitment in omental milky spots is triggered at 4 h after surgery, whereas sham-operated controls showed only little signs of inflammation (Fig. 3a and Supplementary Fig. 4a). To investigate the spread of abdominal bacteria, fluorescently labelled E. coli were injected i.p. Confocal imaging of the omentum after 2 h incubation revealed their presence predominantly in milky spots (Fig. 3b). Recruited neutrophils could be observed in close vicinity to milky spot HEVs and largely colocalize with E. coli (Fig. 3b, Supplementary Movie 9). Flow cytometry showed that CD11b^+^F4/80^+^-macrophages but also CD11b^+^Ly6G^+^-neutrophils can phagocytose bacteria (Fig. 3c). Blocking L-selectin or PNAd abrogates neutrophil phagocytosis whereas macrophage function, assessed at 2 h after E. coli injection, is unaffected in both peritoneal lavage (Fig. 3c) and in omentum extracts (Supplementary Fig. 4c). Next, we injected a sub-lethal dose of E. coli i.p. and assessed sepsis using a murine sepsis score^17^. While control mice developed mild clinical signs over a 2-h time course, blocking PNAd or L-selectin disabled the protective neutrophil response (Supplementary Fig. 4d) which was associated with pronounced sepsis starting ∼45 min after E. coli injection (Fig. 3d). The severe clinical phenotype was associated with greatly increased bacterial loads in the omentum, peritoneal lavage and blood (Fig. 3e). Bacteremia was increased 50 to −100-fold in L-selectin blocked mice. The clinical sepsis score was positively correlated with bacterial burden of the omentum (Fig. 3f). This data suggests neutrophil recruitment via omental milky spot HEVs to be critical for abdominal pathogen clearance and preventing early onset of sepsis.

## Discussion

The outstanding capability of the greater omentum to resolve inflammation and fight bacteria has been clinically appreciated for many decades but the underlying pathomechanisms remain mostly unknown^18^. We report here that a hyperacute phase of mono- and polymicrobial peritoneal inflammation induces rapid neutrophil influx into the omentum by means of milky spot HEVs.

The spatial and temporal molecular pattern of leucocyte- and endothelial-expressed adhesion molecules, chemokines and other regulatory proteins defines the quality and quantity of local cell recruitment. Organ-specific variations of the adhesion cascade such as in liver, brain and lung have been described^19^,^20^. Several lines of evidence argue for a distinct mechanism of neutrophil recruitment in HEVs. We detected a shorter response time compared with PCV after TNFα stimulation. In fact, the mode of leucocyte diapedesis in HEVs seems to be unique as shown by a mouse model with locked interendothelial junctions^21^. Electron microscopy studies highlighted a discontinuous basement membrane and loose lining of mesothelial cells at milky spots that might facilitate fast egress into the peritoneal cavity^22^,^23^.

Furthermore, the panel of required adhesion receptors for neutrophil extravasation is exceptional. Constitutive lymphocyte homing to secondary lymphatic tissue requires L-selectin/PNAd and LFA-1/ICAM-1 interactions in HEVs for rolling and arrest, respectively^9^,^24^. It is known that neutrophils are also capable of rolling in HEVs at steady state in an L-selectin/PNAd-dependent manner^24^ and we show that this is a prerequisite in inflammatory conditions. These findings confirm previous studies on the impact of L-selectin^25^ and its ligand epitope 6-sulfo sialyl Lewis X (ref. 26) in abdominal inflammation and now identify the molecular and cellular mechanisms. In contrast to lymphocytes, Mac-1 and E-selectin but not LFA-1 critically contribute to neutrophil extravasation. Mac-1 is important for luminal crawling^27^ and blockade of E-selectin or its counterreceptor PSGL-1 fully abrogates the downstream adhesion cascade. Interestingly, intravital microscopy reveals that the transition from crawling to arrest requires E-selectin engagement. This finding corroborates a recent report on constitutive E-selectin transcription in HEVs (ref. 28) suggesting modulatory functions in inflammation. While inside-out signalling to LFA-1 is well documented^29^,^30^, a role of E-selectin in LFA-1-independent arrest remains to be investigated.

A rapid phagocyte response is decisive for successful resolution of infection^31^,^32^. Our study attributes the greater omentum a critical task of particularly rapid neutrophil deployment in the peritoneal cavity via HEV-specific mechanisms. Inhibition of this pathway increases bacterial load in the peritoneum and exacerbates septic peritonitis. The critical time window is also known from clinical settings and referred to as ‘golden hours of sepsis'^33^. Some data hint at an association between omentectomy and higher sepsis rates and mortality in patients^34^,^35^. However, murine peritonitis models do not seem to fully recapitulate human pathophysiology^36^ and clinical studies are needed to definitively address the role of the greater omentum in inflammation and sepsis in humans.

The novel insight of HEVs as a distinct vascular entity for neutrophil exit may be relevant in a number of inflammatory and malignant disorders with formation of tertiary lymphoid organs^37^. In accordance with our findings, neutrophil infiltration around lymph node HEVs with E-selectin expression has been described in conditions with elevated TNFα levels such as granulomatosis and Hodgkin's disease^28^,^38^,^39^. The fact that HEV-mediated neutrophil recruitment relies on a unique pattern of adhesion receptors could possibly pave the way for new therapeutic strategies to modulate inflammation.

## Methods

### Animals

*C57BL/6J* (Jackson Laboratories), *LysM-eGFP* (ref. 13; kindly provided by D. Vestweber), Mac-1 knockout^16^ and *Ly6G-tdTomato* mice^14^ (kindly provided by M. Gunzer; all transgenic strains on C57BL/6J background) of both genders were used at the age between 8 and 30 weeks. Animal breeding and experiments were conducted according to the German Animal Welfare guidelines and were approved by the Animal Ethics Committee of the University of Muenster. E. coli and CLP peritonitis experiments were performed at the La Jolla Institute for Allergy and Immunology with the approval of the institutional Animal Care Committee. Peritonitis experiments on Mac-1 knockout mice were performed in the Department of Pathology, Brigham and Women's Hospital.

### Reagents

The following anti-mouse monoclonal antibodies were purchased from BD Bioscience: anti-L-selectin (clone MEL-14, #553147), anti-PSGL-1 (clone 4RA10, #557787), anti-CD11b (clone M1/70, #553308), anti-E-selectin (clone 10E9.6, #550290) and anti-P-selectin (clone RB40.34, #553741). Anti-CD11a (clone 17.4, #22850110) and anti-ICAM-1 (clone YNI.7.4, #22270540) were purchased from Immunotools. Anti–Integrin β7 (clone FIB504, #14-5867-82) was bought from eBioscience. A polyclonal antibody against mouse panlaminin was purchased from Sigma Aldrich (#L9393). Recombinant mouse TNFα, anti-mouse MECA-79 (2-3 μg for labelling, 20 μg for blocking) and PECAM-1 (10 μg for labelling) antibodies were purified from hybridomas (provided by V. Wixler, E. Butcher and D. Vestweber, respectively). Antibody were labelled using amine-reactive fluorescent DyLight 488, 550 and 650 kits (Thermo Fisher). For confocal imaging, the secondary antibodies goat anti-rabbit DyLight 649 (Dianova, 2 μg ml^−1^) and donkey anti-rat Alexa Fluor 555 (Abcam, 2 μg ml^−1^) were used.

### Peritonitis models

Acute peritonitis was induced by i.p. injection of TNFα (500 ng) in 500 μl PBS. In some experiments, blocking antibodies (40 μg in 100 μl PBS) were administered intravenous (i.v.) 15 min before. After 30, 60 or 120 min animals were euthanized by CO_2_ and the abdominal cavity was flushed with 5 ml cold PBS. Subsequently, the omentum was extracted for further analysis. Alternatively, LPS (25 μg, Sigma) was used with an incubation time of 12 h.

The CLP model was performed according to a published protocol with a sub-lethal mortality^40^. We confirmed the low-mortality time course of this protocol (Supplementary Fig. 4e). Briefly, anaesthesia was induced using isoflurane and buprenorphine was administered subcutaneously for post-operative analgesia. After a 1-cm midline incision of the abdomen the caecum was exteriorized. At 1 cm from the distal end, the caecum was ligated using 2-0 suture and perforated using a 27 gauge needle (both sides of the caecum). For short time points (<4 h), the animal was kept in anaesthesia after the operation until euthanasia and subsequent analysis. Sham operation as control did not include ligation and perforation.

For live E. coli experiments, non-pathogenic DH5α were cultured in lysogeny broth medium overnight. Before each experiment, E. coli were incubated for 2 h in fresh medium to induce log phase. After i.v. administration of blocking antibody or vehicle, 1 × 10^8^ E. coli were injected i.p. in 300 μl PBS and clinical signs of sepsis were assessed using a murine sepsis score with a maximum of 28 points^17^. After 2 h the peritoneal lavage, the omentum and blood (in EDTA) was obtained and plated on lysogeny broth agar plates in serial dilutions. Following 24 h of incubation at 37 °C, the number of colonies was determined and expressed as mean of the diluted series.

For imaging studies, heat-inactivated fluorescently labelled E. coli (Bioparticles) were purchased from Molecular Probes. Particles (6 × 10^7^) were diluted in 500 μl PBS and injected i.p. After 2 h, peritoneal lavage and omentum were harvested for confocal microscopy or flow cytometry analysis.

### Intravital microscopy

Mice were injected with fluorescently labelled antibodies i.v. and anaesthetized by i.p. injection of ketamine (125 mg kg^−1^) and xylazine (12.5 mg kg^−1^). In some experiments a carotid catheter was inserted to readminister anaesthesia, inject antibodies or take blood samples. All antibodies used in vivo were azide-free. After removal of abdominal fur, a 1–1.5-cm median incision was done at the linea alba of the upper abdomen to exteriorize the greater omentum. It extends from the greater curvature of the stomach and the spleen and can be distinguished from mesenterial fatty tissue by its lighter appearance. Gentle handling with cotton-wool tips is essential to avoid tissue damage and bleeding was electrocautered if necessary. A custom-made microscopic stage allowed fixation of the omentum between two cover slips. Throughout the experiment the animals were kept warm on a 37 °C heating pad or incubation chamber and the exteriorized specimen was kept moist using heated bicarbonate-buffered saline. Omental inflammation was induced by topically applying TNFα (2.0 μg ml^−1^) in PBS. To visualize HEVs only low doses of MECA-79 antibody (2–3 μg per mouse) were injected. 200 kDa FITC- or rhodamine-dextran was used as blood tracer. Videos were recorded using either a Axio Scope.A1 microscope (Zeiss) at × 10 (numerical aperture 0.25) or × 20 (numerical aperture 0.45) equipped with stroboscopic epifluorescent illumination (Colibri 2, Zeiss) or an upright spinning disc microscope (Zeiss Imager Axio Examiner D1). After imaging, mice were euthanized in anaesthesia and the omentum was extracted and fixed in 2% paraformaldehyde for further analysis.

Data analysis and cell tracking was done using Fiji^41^. Where indicated, movies were denoised via total variation denoising (according to ref. 42 with *β*=0). All video frames were rigidly aligned based on minimizing the so-called L1-distance between them. Colour channels with strong photobleaching but no other temporal change were replaced by the first frame to improve their visibility.

### Omentectomy

Mice were anaesthetized as described above. A 1-cm incision was made in the upper linea alba to expose the greater curvature of the stomach. The omentum was clamped on both most lateral ends using micro clips (0.8 × 5 mm), ligated and subsequently removed. Great care was taken to avoid bleeding. Next, 500 ng TNFα in 500 μl PBS were applied into the peritoneal cavity and the abdominal wall was closed up. After 1 h, the abdominal cavity was lavaged with 2 ml cold PBS. Sham operation consisted of abdominal incision and gentle manipulation of the omentum.

### Flow cytometry

Extracted omenta were meshed using a 70-μm strainer and washed using PBS. Cells were stained using standard flow cytometry protocols with a combination of anti-CD11b-FITC or -APC, anti-Ly6G-FITC or -APC, anti-F4/80-FITC or –APC and CD45-PerCP (1:200 dilution, all from BD Bioscience). Cell viability of omenta and lavage were determined using ghostdye stain (Tonbo) and was >85%.

### Confocal microscopy

Omenta were isolated, fixed in 2% PFA for 1 h, blocked with 5% foetal calf serum for 30 min and incubated with primary antibodies in PBS at 4 °C. Specimens were subsequently stained with the secondary antibodies for 4 h at 4 °C. Whole mount tissues were examined using a Zeiss LSM 700 confocal microscope equipped with a Hamamatsu ORCA–ER CCD camera. Images were analysed using Volocity software (ImproVision).

### Electron microscopy

Milky spots were manually dissected from the omentum, fixed, dehydrated and embedded in epon resin using standard protocols. Sections (60 nm thin) were cut on a microtome and stained with uranyl acetate and lead citrate. Images were taken using a Philips CM10 electron microscope. Milky spots were identified by the high density of parenchymal leukoytes. HEVs showed a higher cell morphology and were luminally populated with mononuclear cells.

### Statistics

Data were analysed using Prism software (GraphPad). Unpaired Student's *t*-test or two-way analysis of variance with Bonferroni posthoc correction was used to compare conditions. Data are expressed as mean±s.d. *P<0.05, **P<0.01, ***P<0.001.

## Additional information

**How to cite this article:** Buscher, K. *et al*. Protection from septic peritonitis by rapid neutrophil recruitment through omental high endothelial venules. *Nat. Commun.* 7:10828 doi: 10.1038/ncomms10828 (2016).

## Supplementary Material

Supplementary Information Supplementary figures 1-4 and Supplementary References

Supplementary Movie 1 Blood-borne neutrophils in the greater omentum preferentially interact in the milky spot microcirculation during early inflammation. Intravital microscopy recording of the greater omentum of a LysM-eGFP mouse topically treated with TNFα 20 min prior to imaging. Brightfield and GFP channels were recorded to show the milky spot and interacting GFPhigh neutrophils, respectively, and the circulation is highlighted in red using a blood tracer. Scale bar 50 µm.

Supplementary Movie 2 Neutrophil interaction in milky spot high endothelial venules. HEVs within a milky spot are visualized using the MECA-79 antibody (red) in a LysM-eGFP mouse (neutrophils in green) after topical TNFα treatment for 20 min. Blood tracer in blue. Scale bar 40 µm.

Supplementary Movie 3 Milky spot high endothelial venules as neutrophil exit site. 4D spinning disc intravital microscopy of a LysM-eGFP mouse treated with TNFα i.p. for 60 min. Labeled low dose MECA-79 antibody (red) was injected to mark HEVs. Maximum intensity projection. Neutrophils are GFPhigh. Scale bar 40 µm.

Supplementary Movie 4 HEV-mediated neutrophil recruitment in Ly6G-reporter mice. Ly6G-tdTomato mice were used as neutrophil reporter (red) and omental inflammation was induced by TNFα 45 min prior to imaging. High endothelial venules are labeled by MECA-79 (green) and a blood tracer outlines the microcirculation. Video was corrected for noise, bleaching of the green channel and motion artefacts. Scale bar 50 µm.

Supplementary Movie 5 HEV-mediated extravasation is faster than in postcapillary venules. Long term intravital video recording in low magnification of the greater omentum of a LysMeGFP mouse treated with TNFα at time point 0 min showing a milky spot (left) and a conventional postcapillary venule (right) in the same field of view over a time course of 105 min. Only GFP channel is shown. Milky spots of LysM-eGFP mice have a low background due to GFPlow macrophages. Neutrophils are GFPhigh. See Fig. 1h and Supplemental Fig. 2b for more information. Scale bar 100 µm.

Supplementary Movie 6 Effect of PNAd blockade in HEVs. High magnification video of a TNFα-inflamed milky spot HEV in a LysM-eGFP mouse treated with blocking PNAd antibody (50 µg labeled MECA-79, red). Neutrophils are green. Scale bar 30 µm.

Supplementary Movie 7 Effect of E-selectin blockade in HEVs. High magnification video of a TNFα- inflamed milky spot HEV in a tdTomato-Ly6G mouse treated with blocking E-selectin (CD62E) antibody. Neutrophils are green, blood tracer in blue. Scale bar 50 µm.

Supplementary Movie 8 Effect of Mac-1 blockade in HEVs. Video of a TNFα-inflamed milky spot in a tdTomato-Ly6G mouse treated with blocking Mac-1 (CD11b) antibody. Neutrophils are in green, blood tracer in blue. Each time point is a merge of 10 consecutive images (each 200 ms apart) resulting in green streaks for rolling cells. Scale bar 50 µm.

Supplementary Movie 9 Neutrophil phagocytosis of E.coli in milky spots. Fluorescent E.coli were i.p. injected using LysM-eGFP mice and the omentum was intravitally imaged after 2 hour incubation. Maximum intensity projection of a 30 µm z-stack. E.coli are red, neutrophils are GFPhigh. Scale bar 10 µm.
