# Supplementary material for: Protection from septic peritonitis by rapid neutrophil recruitment through omental high endothelial venules
Source: Nat Commun. 2016 Mar 4;7:10828. doi: 10.1038/ncomms10828 (PMC4785224; doi:10.1038/ncomms10828)
Supplement: Supplementary Information — Supplementary figures 1-4 and Supplementary References [file ncomms10828-s1.pdf]

Supplementary Fig. 1

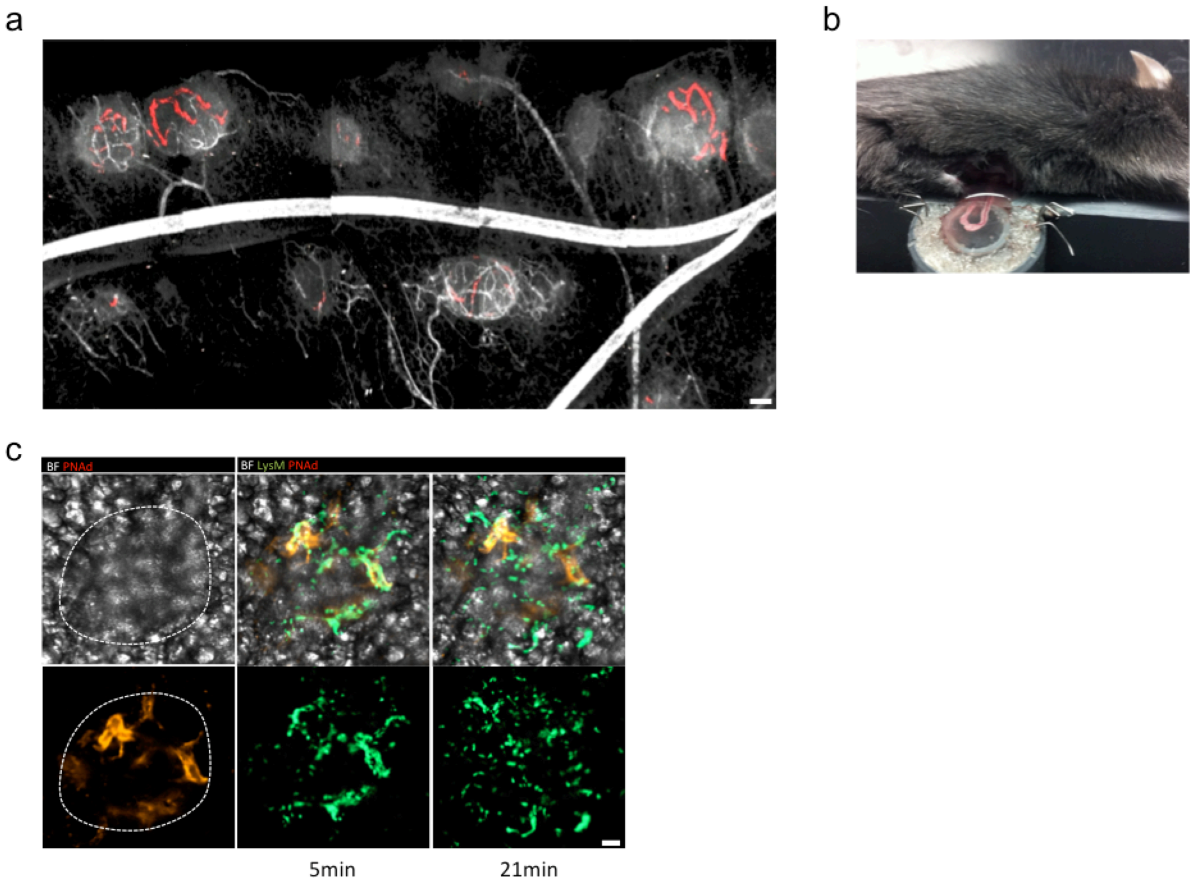

Milky spot HEVs are preferred neutrophil exit sites in abdominal inflammation. a) Stiched maximum projection image acquired by confocal microscopy showing PECAM-1- (white) and PNAd-staining (red) in the greater omentum. Note the presence of HEVs in each milky spot. Scale bar 50 µm. b) Operation technique to exteriorize and image the greater omentum intravitally in an anaesthetized mouse. c) Intravital video recording of a milky spot showing brightfield characteristics and MECA-79 (red) staining of milky spots (dashed line) and HEVs. The center and right panels depict early and late accumulation of GFP^{high} neutrophils after TNFα treatment, respectively. Scale bar 40 µm.

Supplementary Fig. 2

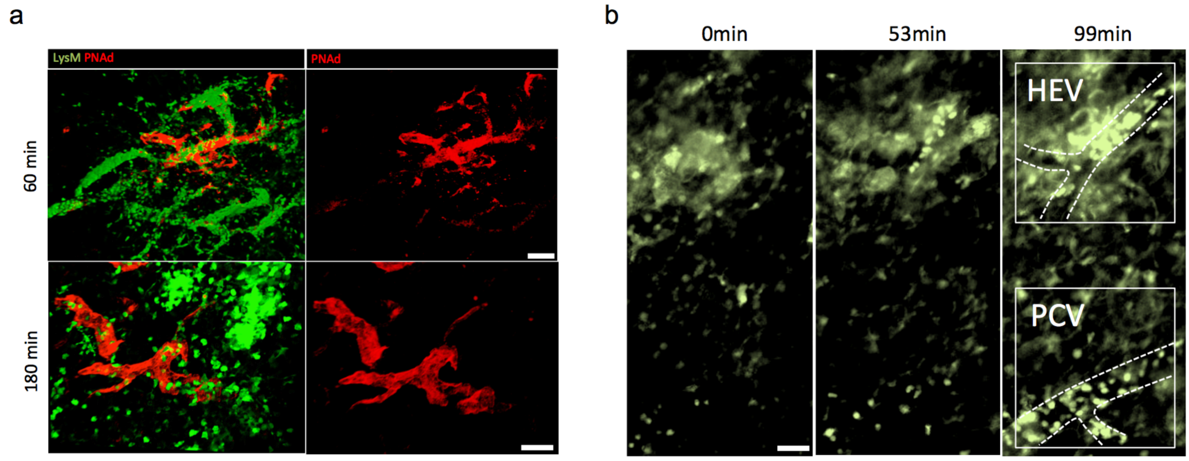

HEV-mediated neutrophil exit is faster than in conventional postcapillary venules. a) Confocal images of milky spot PNAd$^+$-HEVs and adjacent PNAd$^-$-vasculature in LysM-eGFP mice after exposure to TNFα. At 60 min, GFP^{bright} neutrophils were mostly located intraluminally in PNAd$^-$ venules compared to PNAd$^+$ HEVs indicating early exit in HEVs. After 180 min, neutrophil extravasation was also evident in PNAd$^-$ vasculature. Scale bar 40 μm. b) Raw video data of Figure 1g showing that the temporal dynamics of leukocyte recruitment in PNAd$^+$ HEVs is faster than in PNAd$^-$ postcapillary venules. The field of views to determine the fluorescence intensity are indicated on the right panel (200x200 μm). 3 time points are depicted here from a 105 min video (1 frame/4s). Note the presence of GFP^{low} macrophages in the milky spot (left). For data analysis, background fluorescence of time point 0 min was subtracted. Scale bar 25 μm.

Supplementary Fig. 3

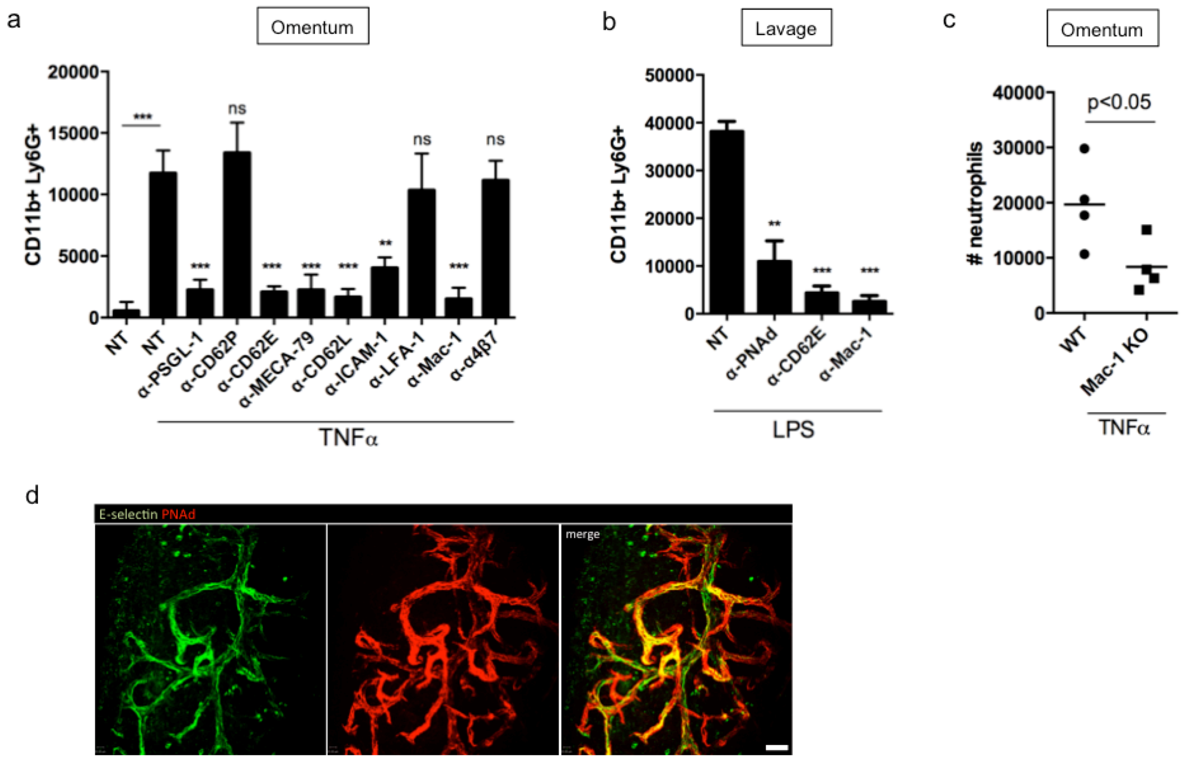

Effect of adhesion receptor blockade on neutrophil exit in milky spots. a) Impact of different receptor blocking antibodies on $CD11b^{+}Ly6G^{+}$ neutrophil extravasation into the omental parenchyma after 1h TNFα-induced peritonitis. n=6 of at least 3 independent experiments except α4β7 with n=3. Data as mean total cell number ± s.d. Except for NT without TNFα, statistical significance is indicated relative to NT with TNFα. b) Impact of blocking anti-E-selectin, anti-Mac-1 and anti-PNAd antibodies on neutrophil migration into the peritoneal cavity in LPS-induced peritonitis. n = 3 in all groups. Data as mean ± s.d. c) TNFα was injected i.p of age/gender matched Mac-1 KO or wild type mice. After 1h, neutrophil recruitment in the greater omentum was assessed by flow cytometry by enumerating neutrophils as $CD45^{+}Gr\text{-}1^{+}7/4^{+}$ cells. Shown are 4 animals per group of one experiment and representative of two experiments. d) Confocal micrographs depicting E-selectin expression in milky spot HEVs after 1h TNFα incubation. Representative of more than 6 milky spots in 3 independent experiments. Scale bar 50 μm.

Supplementary Fig. 4

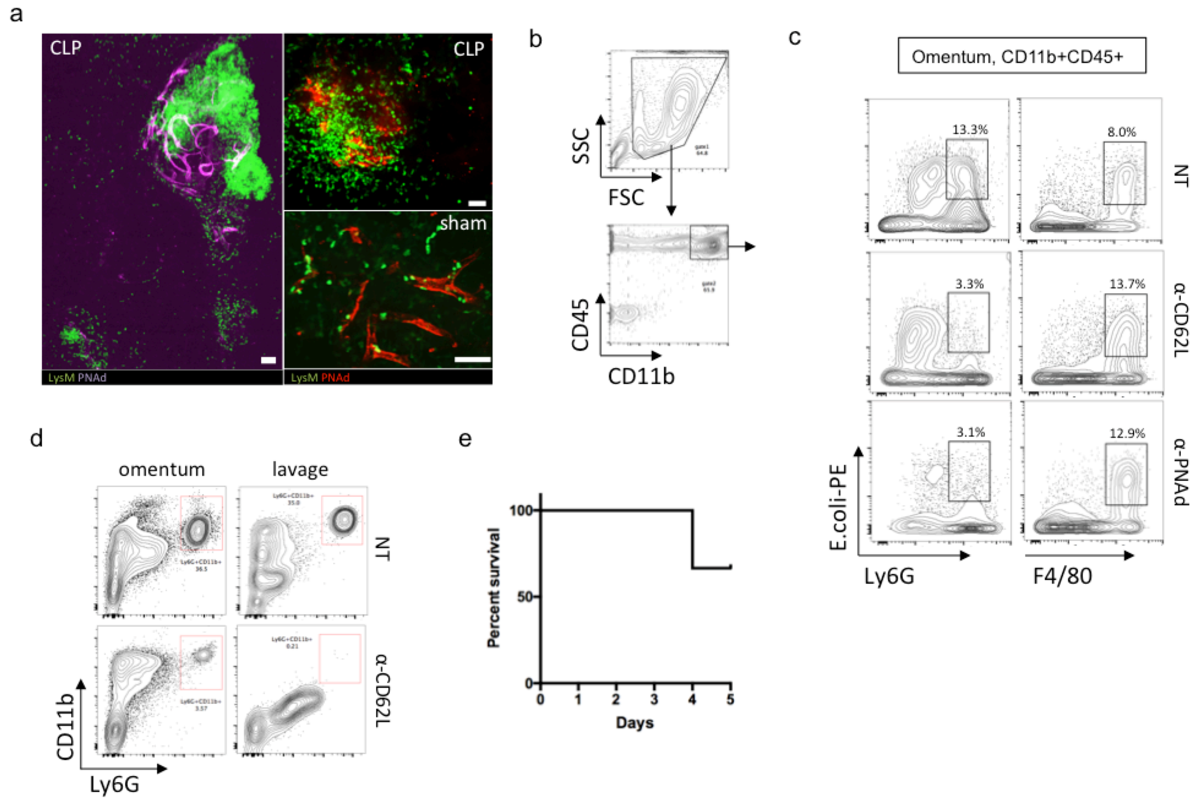

Effects of peritoneal bacterial infections on omentum and lavage. a) Polymicrobial peritonitis induced by cecal ligation and puncture (CLP) shows substantial LysM-eGFP neutrophil extravasation at PNAd^{+} HEVs in omental milky spots (red/magenta) 4h after surgery, whereas only few neutrophils (green) can be observed in sham-operated controls. Scale bar 50 µm (right) and 100 µm (left). Left image is a stiched confocal image. Representative of 3 experiments. b) Gating strategy for Fig. 3b and Suppl. Fig 4b. c) Flow cytometry data of the omental parenchyma showing colocalization of fluorescently labeled E.coli and CD11b^{+}F4/80^{+} macrophages or CD11b^{+}Ly6G^{+} neutrophils 2h after infection. L-selectin or MECA-79 blockade reduces neutrophil phagocytosis whereas macrophage colocalization is unaffected. Similar data has been obtained for the peritoneal lavage (Fig. 3b). Gating strategy in panel b. d) Flow cytometry data of CD11b^{+}Ly6G^{+} neutrophils in the peritoneal lavage or omental extracts of mice i.p. treated with E.coli for 2h and the effect of L-selectin blockade prior to infection. e) Sub-lethal CLP using a 27G needle was performed as previously described in detail [1] and low mortality was confirmed. n = 3.

References

1. Cuenca, A. G., Delano, M. J., Kelly-Scumpia, K. M., Moldawer, L. L. & Efron, P. A. Current Protocols in Immunology: Cecal Ligation and Puncture. *Curr. Protoc. Immunol.* (2010). doi:10.1002/0471142735.im1913s91
